# The Challenges of Diagnostic Imaging in the Era of Big Data

**DOI:** 10.3390/jcm8030316

**Published:** 2019-03-06

**Authors:** Marco Aiello, Carlo Cavaliere, Antonio D’Albore, Marco Salvatore

**Affiliations:** 1 IRCCS SDN, Via Gianturco 113, Napoli 80143, Italy; ccavaliere@sdn-napoli.it (C.C.); direzionescientifica@sdn-napoli.it (M.S.); 2 Biocheckup SRL, Napoli 80121, Italy; adalbore.biocheckup@gmail.com

**Keywords:** Big data, Diagnostic Imaging, Radiology, Radiomics, Human Connectome, Brain Connectivity, Anthropometry, Simulation

## Abstract

The diagnostic imaging field has undergone considerable growth both in terms of technological development and market expansion; with the following increasing production of a considerable amount of data that potentially fully poses diagnostic imaging in the Big data in the context of healthcare. Nevertheless, the mere production of a large amount of data does not automatically permit the real exploitation of their intrinsic value. Therefore, it is necessary to develop digital platforms and applications that favor the correct and advantageous management of diagnostic images such as Big data. This work aims to frame the role of diagnostic imaging in this new scenario, emphasizing the open challenges in exploiting such intense data generation for decision making with Big data analytics.

## 1. Introduction

The diagnostic imaging field undergoes considerable growth in terms of technological development, innovation, and market expansion, with the following increasing production of a considerable amount of data that potentially fully poses diagnostic imaging in the context of Big data. 

Big data refers to extremely complex datasets that are characterized by typical attributes that have been defined over time all characterized by the initial letters “V”: volume, which refers to the sheer number of data elements within these extremely large datasets; variety, which describes the aggregation of data from multiple sources; and, velocity, which refers to the high speed at which data is generated, constitute the first three “Vs” [1]; subsequently, veracity, which describes the inherent uncertainty in data [2], and value, which refers to the worth of the data being extracted have been included [3]. Alternative, less direct, definitions refer to the unconventional capability that is required for managing and analyzing huge data volumes; therefore, Big data analytics methods are specifically designed to overcome this challenge.

Rather than the representation by several Vs, Wu et al. [4] characterized Big data more effectively, by the so-called HACE (Huge, Autonomous, Complex, Evolving) theorem: Big data employ large and heterogeneous data volume, including autonomous sources with distributed and decentralized controls, and seek to explore complex and evolving relationships among data.

It is worth pointing out that major corporations, such as Facebook, have embraced the explosion of information (billions of photos and millions more each day) by committing to open-source projects and information sharing.

In the healthcare, Big data encompasses objective and subjective findings, parametric and non-parametric measures (e.g., diagnosis, demographics, treatment, prevention of disease, illness, injury, and physical and mental impairments), derived from different sources using incongruent sampling. These data are structured, focusing on genotype, proteomic data or clinical scores, or, on large part, unstructured, including memos, clinical notes, prescriptions, medical imaging, lifestyle, environmental, and health economics data [5]. 

Radiologists and researchers should strive to make progress toward harnessing Big data in imaging, which has the potential to lead to advanced clinical support, personalized diagnostic and prognostic tools, and the ability to optimize individual patient outcomes in ways that were previously not possible [6]. Big data analytics refers to the analysis of the large, identifying clusters and correlation between datasets, as well as developing predictive models through the use of data mining techniques [7].

Following, the five main characteristics that should be respected in the configuration of Big data in diagnostic imaging field are described.

### 1.1. Volume

The volume refers to the quantity of Big Data in healthcare, which is estimated to dramatically increase until the order of zettabytes (1021) by 2020. According to the Stanford Medicine 2017 Health Trends Report, the sheer volume of health care data is growing at an astronomical rate: 153 exabytes (one exabyte = one billion gigabytes) were produced in 2013 and an estimated 2314 exabytes will be produced in 2020. Today, data that surround most of our lives are collected and stored; data scientists are exploring applications that could harness this information and make it valuable [5]. Of this amount, diagnostic imaging, together with genomic information, represent a large part. Note that, according to Eurostat reporting, each year, one person in ten in Europe undergoes computed tomography (CT) imaging, one in 13 undergoes magnetic resonance (MR) imaging and one in 200 positron emission tomography (PET) imaging. These rates accurately reflect the law of increase of computational power (Moore’s law); however, they are expected to significantly underestimate the actual rate of increase of data acquisition [5].

### 1.2. Variety

The variety refers to the different types of healthcare Big data collected, including their heterogeneous characteristics [8] and structured and unstructured nature of medical data [7]. In effect, advances in technology continue to create an increasing ability to multiple measurements on a single sample. This may result in millions of measurements being made concurrently, often combining different technologies to provide simultaneous measures of physiological and clinical features, including measures of disease activity, progression, and related metadata [8]. 

For example, modern imaging techniques perfectly demonstrate the complexity of the obtainable dataset, encompassing different imaging techniques, each with several advantages and drawbacks. Even if only one of the diagnostic techniques is considered, many disparities can rise from different vendors, scanner technology, acquisition parameters, and contrast agents used. In addition to the imaging data, the majority of brain mapping studies include complex meta-data, clinical information, genetics data, biological specimens, meta-data, and other auxiliary observations [5]. 

### 1.3. Velocity

**Figure 1:**
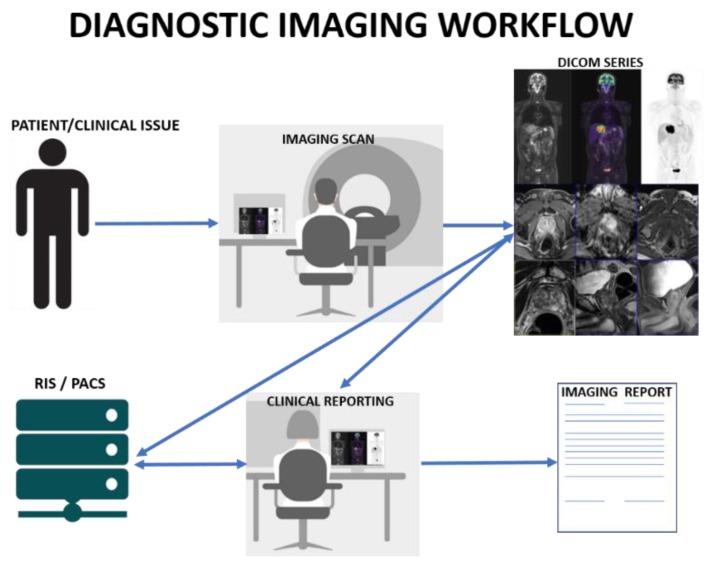
Diagnostic Imaging Workflow. Diagnostic images are acquired through an imaging device, stored in a standard picture archiving and communication (PACS) and radiological information system (RIS) and therefore visually inspected on a digital imaging and communications in medicine (DICOM)/PACS viewer by a specialist (usually radiologists or nuclear physicians), who produces a structured or unstructured report representative of the clinical outcome of the examination.

The velocity is the speed of data generation (i.e., real-time patient data ) as well as data collection [3]. Figure 1 depicts a typical workflow that is related to the diagnostic imaging procedure. Every day, millions of patients undergo diagnostic imaging procedures, which consist of a clear workflow in which information is acquired through an imaging device, stored in a standard picture archiving and communication (PACS) system, and therefore visually inspected on a digital imaging and communications in medicine (DICOM)/PACS viewer by a specialist (usually radiologists or nuclear physicians), who produces a structured or unstructured report representative of the clinical outcome of the examination. 

Beyond this conventional workflow, new evolving procedures must be considered, which include cloud computing of complex data, teleradiology from a site far from the acquisition scanner, or simply second look activities finalized to check quality and/or advice for specialist reports. All of these procedures require the transfer of thousands of images to other sites and it must guarantee download/upload velocity, data integrity, and security, which all must accord for the local privacy laws.

### 1.4. Veracity

The veracity refers to sources that influence accuracy, such as inconsistencies, missing data, ambiguities, deception, fraud, duplication, spam, and latency [9]. Veracity and data quality issues are fundamental issues in healthcare, because life or death decisions depend on the reliability of information. On the other hand, uncertainty is one of the intrinsic attributes of Big data and the application to the healthcare domain must deal with this critical dichotomy. 

### 1.5. Value

The value represents cost–benefit to the decision maker through the ability to take meaningful action that is based on insights derived from data [9]. To fully understand the actual value of diagnostic images and, therefore, the relative potential in Big Data analytics, it is essential to recognize that the information that is carried by diagnostic images goes well beyond that analyzed by a specialist and, therefore, strictly necessary to provide a diagnostic answer. With this in mind, it is necessary to introduce the recent research trends that are extremely promising for the near future.

## 2. Radiomics

**Figure 2:**
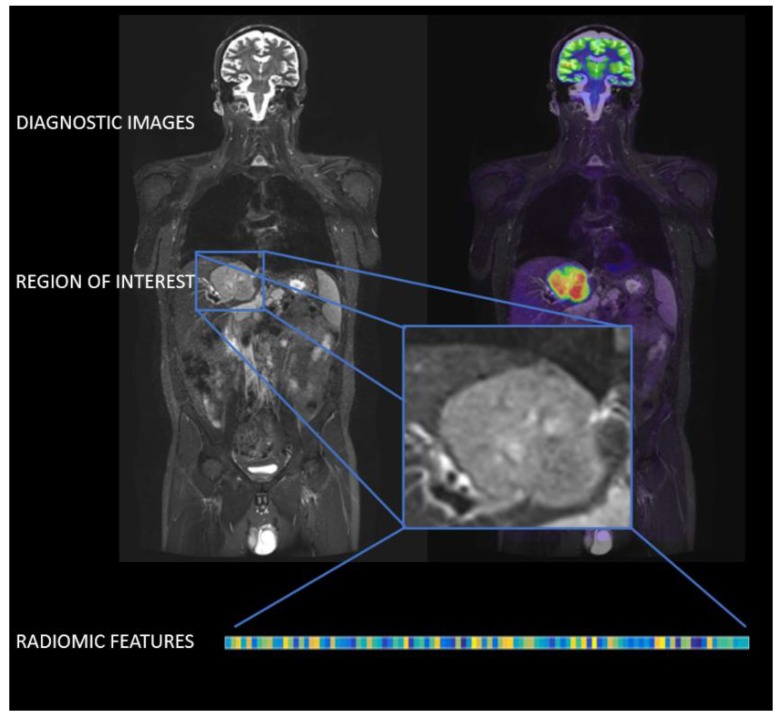
Radiomics workflow. The image shows the three main steps involved in the estimation of radiomic features: magnetic resonance (MR) image acquisition, definition of regions of interest (ROIs), and extraction of numerical descriptors (radiomic features).

The increasing interest in the development of noninvasive diagnostic tools for disease characterization and monitoring, together with the need to capture all of the meaningful information contained in the diagnostic images, give rise to the discipline of radiomics. Radiomics refers to the comprehensive characterization of tumor phenotypes by the extraction of many quantitative features from medical images. This high-throughput extraction of quantitative imaging features is the result of a workflow that is composed of three main steps (Figure 2): image acquisition, definition of regions of interest (ROIs), and extraction of numerical descriptors (radiomic features) [10].

Radiomic analysis exploits sophisticated image analysis tools and the rapid development and validation of medical imaging data and it demonstrates the high potential of using image-based signatures for precision diagnosis and treatment, providing a powerful tool in modern medicine [11].

Radiomics is proving extremely valuable in study the cancer imaging phenotypes, its relationship with underlying gene expression patterns, and reflecting heterogeneous tumor metabolism and anatomy of different cancer types [12,13,14,15,16,17,18]. 

The essence of radiomics is the high-throughput extraction of quantitative image features to characterize ROIs. As a result of features extraction, up to hundreds of features are obtained from a single image. The set of features can be divided into a number of families, of which comprise intensity-based, intensity histogram-based, intensity-volume histogram-based, morphological features, local intensity, and texture matrix-based features [19]. 

Radiomics provides numerical descriptors of the ROIs that can be directly used for the creation of Big data analytics models to support the clinical management of the patients [20].

## 3. Connectomics

Currently, one of the main challenges in modern neuroscience is gaining an understanding of how brain activity generates behavior and, in more detail, how neural information is segregated and integrated. Anatomical and physiological studies support the idea that cognitive processes depend on interactions among distributed neuronal populations and brain regions [21]; connectomics refers to the structural and functional characterization of those interactions. Neuroimaging plays a crucial role in connectomics, and magnetic resonance (MR) imaging (MRI) is commonly used as a reference tool for the investigation of structural and functional connectivity [22,23,24,25]. 

**Figure 3:**
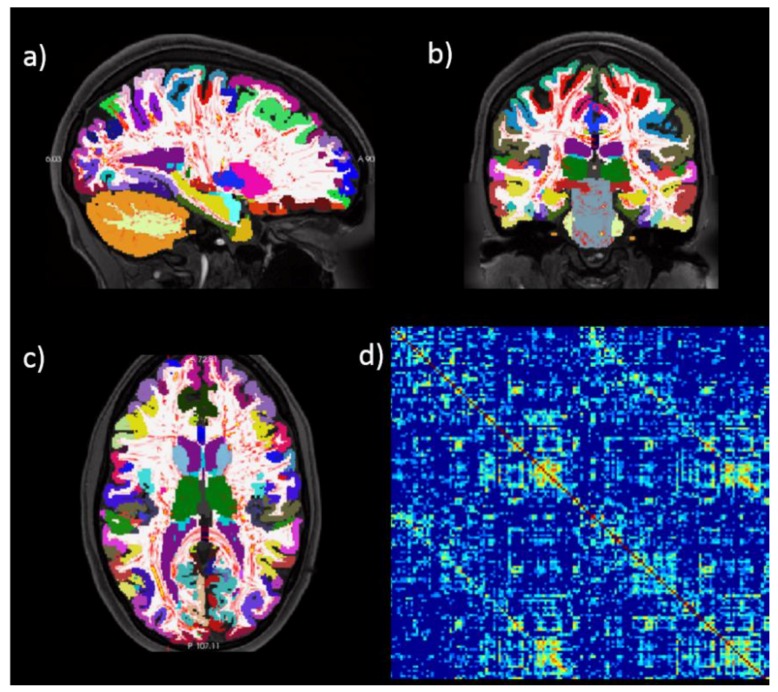
Human connectome estimation. The image shows an example of connectome generation from MR images: (**a–c**) MR anatomical images can be segmented into several meaningful regions, diffusion imaging provides an estimation of axonal connections linking each pair of parcels (red tracts) for the construction of the structural connectome matrix (**d**).

The connectome can be defined as the complete, point-to-point spatial connectivity map of neural pathways in the brain [26] revealed by diffusion imaging and tractography techniques. MR can provide a coarse estimation of the human connectome at the subjective level; Figure 3 shows an example of connectome generation from MR images: MR anatomical images can be segmented into several meaningful regions (the so-called brain parcellation procedure [27]), diffusion imaging provides an estimation of axonal connections linking each pair of parcels for the construction of the structural connectome matrix [28]. 

By combining genomic, phenotypic and functional connectome measures, establishing an individual fingerprint that can reliably identify patients with brain disorders from healthy people and optimize individual treatment plans, may be possible in the future. The progress of personalized medicine is expected to be facilitated by these crucial concepts and research [29].

The study of human connectome has significant repercussions on domains that go beyond the purely clinical, influencing the decision-making process up the socio-economic domain. For example, [30] shows that the local architecture of white matter fascicles reflects a meaningful portion of the variability of social, biological, and cognitive attributes between subjects; anatomical variability in prefrontal cortices and intra-cortical myelin, as revealed by MRI, is linked to individual differences in the socio-cognitive dispositions that are described by the five-factor model of personality [31,32]; furthermore, individual differences in specific facets of decision making competence are mediated by functional connectivity [33]; even, intelligence, as estimated by IQ, is linked to morphological connectomes that are generated by anatomical MRI [34].

These findings can greatly demonstrate the impact of connectomics in the Big data scenario.

## 4. Anthropometry and Simulation

The medical image community has always been fascinated by the possibility of creating simulated or synthetic data upon which to understand, develop, assess, and validate image analysis and reconstruction algorithms. From very basic digital phantoms, all the way to the very realistic silico models of medical imaging and physiology, the community has progressed enormously in the available techniques and their applications. For instance, mechanistic models (by imaging simulations) that emulate the geometrical and physical aspects of the acquisition process have been used now for a long time. Advances on computational anatomy and physiology have further increased the potential of such simulation platforms by incorporating more realism to the simulations that can rely on complex spatio-temporal dynamics due to changes in anatomy, physiology, disease progression, patient, and organ motion, etc. [35].

In the 17th century Elsholtz introduced the term introduced Anthropometria as a quantitative approach to seek information concerning variations and changes in the forms of organisms that described the relationship between the human body and disease. He proposed that the use of anthropometry constituted a valuable measurement strategy for different fields, such as medical practices, physiognomy, the arts, and ethics [36]. 

The importance of anthropometry in the Big data scenario lies in the fact that it allows for making considerations and, more importantly, decisions, which go beyond the clinical-diagnostic field, producing information linked to socioeconomic status and poverty that indicated body size was a signal for the quality of life. Thus, anthropometric methods might be used for social welfare, where factors, such as culture, society, behavior, and the political economy, play an important role in the outcomes of growth and body size [36].

Below, some examples of anthropometry studies based on tomographic imaging are reported:

In [37], a method for obtaining retrospective data on skeletal anthropometry and relating them to the population when subject anthropometry is unavailable is defined; it enables the creation of models that can predict injury in humans of all shapes and sizes.

In the MASALA (Mediators of Atherosclerosis in South Asians Living in America) study, the relationships of anthropometric measures with visceral and subcutaneous fat appear are demonstrated to be similar to other race/ethnic groups, but with weaker non-linearity and heterogeneity by sex [38,39].

When considering that the nose plays a critical role in determining the external appearance of an individual, in [40], researchers studied the craniofacial anthropometrics by CT scanning, providing objective and quantitative data that can help surgeons to plan cosmetic procedures for the nose. Another study [41], established and reported normal anthropometric orbital measurements in a pediatric population using fine-cut craniofacial CT. These measurements are essential for evaluating children with craniofacial anomalies. 

The characterization of the mechanical properties of arterial tissues usually involves an invasive procedure that requires tissue removal. A non-invasive method to perform a biomechanical analysis of cardiovascular aortic tissue, combining MR imaging and finite element analysis, produced an estimate of the in situ properties of cardiovascular tissue [42].

Another study [19] concluded that the 12th thoracic vertebra is more accurate for sex determination than the first lumbar vertebra in the Egyptian population, which means that bone dimensions are population specific.

## 5. Discussion

Diagnostic imaging data, if appropriately managed, can fully exploit the potential of Big data analytics. In this work, it has been shown that diagnostic images, such as total body scan or amagnetic resonance of the brain, can tell a lot of things, which go far beyond the clinical information, about us.

**Figure 4:**
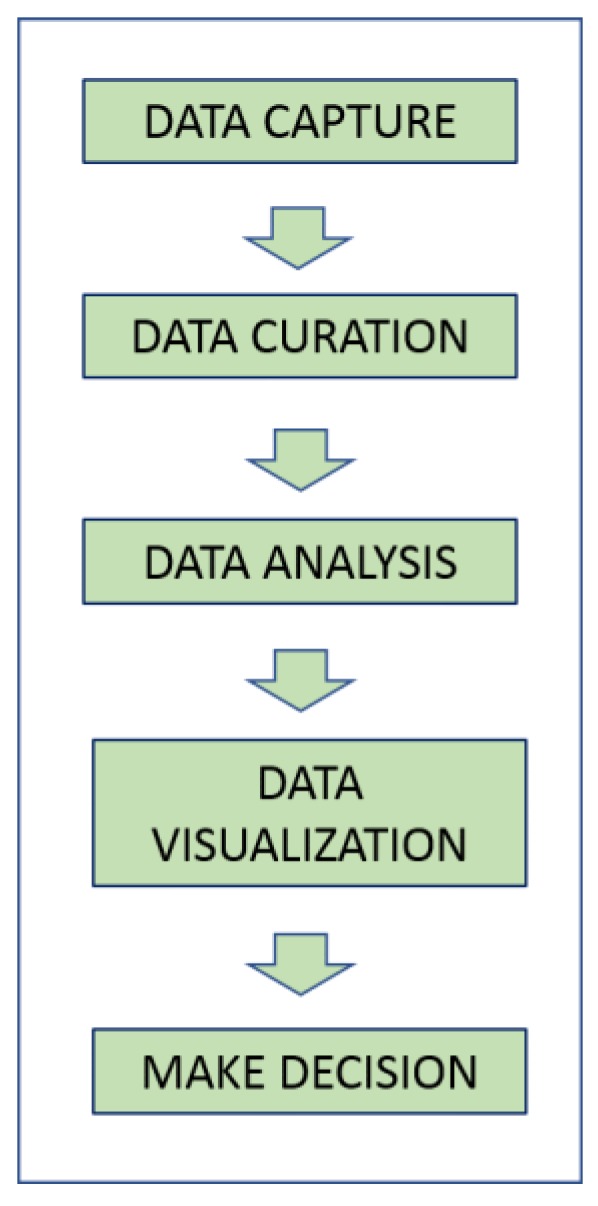
Workflow for decision making in Big data science.

The remarkable value of biomedical images, which can influence decision-making processes well beyond the diagnostic scope, emphasizes the importance of the chain of processes that can lead to the final decision. Figure 4 shows main processes that are involved in processing Big data: data capture, data curation, data analysis, data visualization, and decision making [43]. Each of these steps requires, in turn, suitable decision protocols with the specific aim to meet the Big data science. For example, recalling the definition of Big data, all Vs are strictly interconnected, and it is not enough to make available just a huge amount of data to make decisions with Big data analytics. 

According to the HACE theorem, massive production of imaging data from autonomous and monolithic sources (local diagnostic centers), without distributed and decentralized controls, can dramatically reduce the potential of Big data analytics. Therefore, a primary challenge can be recognized as the need of empower data sharing from single sources of data. Each step of Figure 4 can be potentially critical; if a satisfactory amount of imaging data is acquired and shared worldwide daily, then it is not assumed that all of these data can be used to apply Big data analytics techniques; in this light, the data curation step can constitute a critical bottleneck. 

In the diagnostic imaging, raw information is released as a simple raster of pixels or voxels; on the other hand, a high-level representation (tissues, organs, lesions, etc.) is desirable for estimating numerical descriptors, such as that described for radiomics and anthropometrics fields. To this aim, it is necessary to obtain as much clinical annotations as possible, in the form of manual segmentations and ROIs delineations by specialists, for the subsequent training of supervised machine learning techniques as well as automated procedures. As matter of fact, this is challenging and should likely concur with the normal radiological workflow that is described in Figure 1; a suitable solution could consist in the implementation of tools that are able to track and log the interactions of the radiologists during the reporting procedures. This issue cannot disregard the use of DICOM standard to enable structured, standardized, and interoperable communication of the clinical annotations [44].

The curation of data is the subject of various initiatives of standardization of acquisition protocols and procedures of annotation of images for various types of data and diseases [19,45,46,47,48], as well as of trustable repository of imaging data, such as imaging biobanks.

Indeed, imaging biobanks, as defined by the European Society of Radiology, are “organised databases of medical images, and associated imaging biomarkers (radiology and beyond), shared among multiple researchers, linked to other biorepositories” [49]. 

The dissemination of imaging biobanks can certainly improve the reliability and discovery of imaging biomarkers, but, on the other hand, can deny the free propagation of uncertain data requested by Big data science, demonstrating a dichotomy that is now well established. 

Recalling Gödel’s incompleteness principle, healthcare data cannot be consistent and complete at the same time. In other words, any computational inference, or decision making, which is based on Big data would be expected to either be reliable within a restricted domain (e.g., time, space) or be more broadly applicable (e.g., cohort or population studies) but less consistent. This dichotomy is also supported by statistical inference rules, where small or large sample sizes depend on the corresponding large or small variances of parameter estimations, respectively [5].

With the opportunities that are created by digital and information revolution, the healthcare industry can exploit the potential benefits of leveraging Big data technology. The predictive nature and pattern-recognition aspects of Big Data analytics enable the shift from experience-based medicine to evidence-based medicine [3].

The analysis of successful industries, such as Google, Netflix, Facebook, and Amazon, reveals that they have free and open access to data, which are willingly provided by the customer and centrally delivered to the company. When compared with industry, for the most part, the situation is different in healthcare [50]. Medical images, which represent deep private personal information, are not openly available; data are usually stored in diagnostic centers, limiting the velocity or volume of data that are required to exploit Big data methods. 

Probably, a suitable strategy is to put the patient at the center of clinical procedures through appropriate digital platforms and communities that could definitively favor a conscious sharing and a valuable centralized confluence of data.

Emerging topics in diagnostic imaging are revealing the need to integrate an unprecedented amount of different specialties. The interdisciplinary work involves the collaboration of physicians (radiologists, radiographers, nuclear physicians, oncologists, and other specialists), not only with physicists and bioengineers, but, increasingly, with computer scientists, biostatisticians, and experts in bioinformatics [50]; therefore, leveraging the interaction and targeted educational programs across technical and clinical specialties can decisively boost the application of Big data Science to diagnostic imaging. 

In conclusion, diagnostic imaging can greatly exploit the potential of Big data analytics for decision making in a wide class of domains; there are still open challenges and barriers concerning data sharing, datacuration, and interdisciplinary education, which require further efforts for the implementation of suitable solutions. It is essential to enable an ordinary confluence of data towards centralized repositories; such Big data should be enriched with proper clinical annotations and released with full awareness of the patient, who should be placed at the center of the diagnostic workflow.
